# A Copper-Based Metal-Organic Framework as an Efficient and Reusable Heterogeneous Catalyst for Ullmann and Goldberg Type C–N Coupling Reactions

**DOI:** 10.3390/molecules201219756

**Published:** 2015-11-27

**Authors:** Wei Long, Wenge Qiu, Chongwei Guo, Chuanqiang Li, Liyun Song, Guangmei Bai, Guizhen Zhang, Hong He

**Affiliations:** 1 Beijing Key Laboratory for Green Catalysis and Separation, Department of Chemistry and Chemical Engineering, College of Environmental and Energy Engineering, Beijing University of Technology, Beijing 100124, China; longwei007@163.com (W.L.); gcwhome@emails.bjut.edu.cn (C.G.); songly@bjut.edu.cn (L.S.); baiguangmei@bjut.edu.cn (G.B.); zhangguizhen@bjut.edu.cn (G.Z.); 2 Department of Applied Chemistry, College of Science, Chongqing Jiaotong University, Chongqing 400074, China; lichuanqiang_cn@163.com

**Keywords:** metal-organic frameworks, C–N coupling, heterogeneous catalysis, copper complex

## Abstract

A highly porous metal-organic framework (Cu-TDPAT), constructed from a paddle-wheel type dinuclear copper cluster and 2,4,6-tris(3,5-dicarboxylphenylamino)-1,3,5-triazine (H_6_TDPAT), has been tested in Ullmann and Goldberg type C–N coupling reactions of a wide range of primary and secondary amines with halobenzenes, affording the corresponding *N*-arylation compounds in moderate to excellent yields. The Cu-TDPAT catalyst could be easily separated from the reaction mixtures by simple filtration, and could be reused at least five times without any significant degradation in catalytic activity.

## 1. Introduction

The *N*-aryl heterocycles are very important structural motifs in biological, pharmaceutical and material science products [1,2,3]. Formation of C–N bonds by Ullmann and Goldberg coupling reactions represents a powerful tool for the preparation of these nitrogen-containing compounds on both laboratory and industrial scales [4,5,6,7]. However the classic Ullmann and Goldberg coupling reactions suffer from drawbacks such as harsh reaction conditions, including high reaction temperatures, extended reaction times, and the use of large amounts of copper. Since two separate research groups achieved an important breakthrough with the discovery of efficient copper/ligand systems that enable these cross coupling reactions to occur under much milder conditions at the beginning of the 21st century [8,9], the formation of C–N bonds by copper-mediated cross-coupling approaches has undergone a renaissance in the last decade [10,11,12]. Many classes of ligands, including diamines [4,13], amino acids [14,15,16], amino alcohols [17,18], diols [19], diethylamine [20], diphosphines [21], phenanthrolines [22,23], pyridine *N*-oxide [24], β-diketones [25,26,27] and metformin [28,29], have been used to promote the copper-catalyzed cross-coupling C–N bond-forming reactions. Moreover, a few metal- and ligand-free [30,31,32], and photoinduced [33,34,35] C–N coupling reactions have also been reported. Meanwhile, a few mechanistic [36,37] and computational studies [38] have been conducted on these copper-mediated cross-coupling reactions in order to understand these intriguing catalytic processes in detail.

Because of the increasing concern regarding environmental impact, the heterogeneous catalysis has received much attention recently due to its advantages of ease of product separation, catalyst recovery and recyclability. Several categories of copper-based heterogeneous catalysts for the Ullmann and Goldberg coupling reactions, including copper compound nanoparticles [39,40,41], inorganic materials (e.g., mesoporous nitrogen-doped carbon [42], SiO_2_ [43,44], fluoroapatite [45] and Fe_3_O_4_ [46,47])—supported catalytic systems, and organic polymer (e.g., polystyrene [48,49,50], chitosan [51] and polytriallylamine [52])-supported catalytic systems, have been reported. In the past decade, metal-organic frameworks (MOFs) have received much attention as catalytic materials in addition to their applications in gas storage and separation due to their unique features, including their crystallinity, porous structure, huge specific surface area and the high density of open metal sites in the framework [53,54,55]. Several studies on the catalytic activity of MOFs with active open metal sites have shown their potential applications in reactions like hydrogenation [56,57], isomerization [58], cyanosilylation [59,60], oxidation [61,62,63,64,65], photocatalysis [66,67], Friedel-Crafts reaction [68,69,70], and condensation reactions [71]. However, the Ullmann and Goldberg type C–O, C–N and C–S coupling reactions over MOFs have barely been explored [72,73,74,75]. Cu-TDPAT [76] based on [Cu_2_(COO)_4_] square paddle-wheel secondary building units (SBUs) with *rht*-topology is highly porous, with a large surface area and pore volume, and should be a good potential copper-based heterogeneous catalyst. Herein, we report the applications of Cu-TDPAT as an efficient and reusable heterogeneous catalyst for Ullmann and Goldberg type C–N coupling reactions.

## 2. Results and Discussion

**Figure 1:**
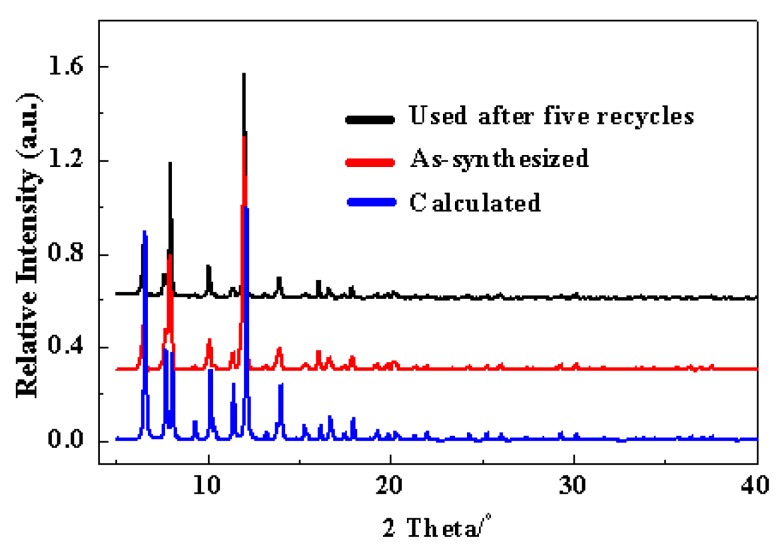
X-ray diffraction patterns for Cu-TDPAT samples, as-synthesized (**red**); and used after five catalysis cycles (**black**).

Cu-TDPAT catalyst was prepared by a solvothermal method according to the reported procedure [76]. The SEM image of Cu-TDPAT sample clearly showed that most of the MOF particles presented polyhedral shapes (Figure S1). The crystal size of the Cu-TDPAT sample was in the range of 10–40 µm. The powder XRD pattern of the as-synthesized Cu-TDPAT (Figure 1) matched well with the published results [76], confirming the formation of the intended crystalline framework. Nitrogen physisorption measurements demonstrated the porosity and stability of Cu-TDPAT after removing the included and coordinated solvents (Figure S2, Table S1). Its BET surface area was 1855 m^2^/g. Thermogravimetric data showed that Cu-TDPAT was stable up to a temperature of 250 °C (Figure S4).

### 2.1. Cu-TDPAT as a Solid Catalyst for the N-Arylation of 5-Methyl-2-(1H)-Pyridone

**Scheme 1:**
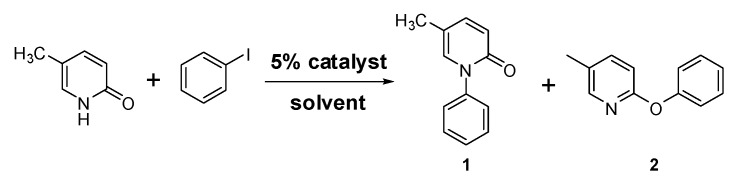
Coupling reaction of iodobenzene and 5-methyl-2-(1*H*)-pyridone.

To test the use of Cu-TDPAT as a catalytic copper complex, iodobenzene and 5-methyl-2-(1*H*)-pyridone were selected as coupling partners in the initial screening of optimal reaction conditions (Scheme 1) as the coupling product of these two substrates, 5-methyl-1-phenyl-2-(1*H*)-pyridone, also known as pirfenidone (**1**), has been a widely used drug for the treatment of idiopathic pulmonary fibrosis (IPF) [77,78].

The experimental results under various conditions are presented in Table 1. It was found that no product was observed after 2 h at 80 °C (Table 1, entry 2), indicating that the coupling reaction proceeded with difficulty at or below this temperature. With increasing temperature, the yields of pirfenidone (**1**) increased gradually (Table 1, compare entries 3, 4, 5). The highest yield of **1** was obtained at 140 °C. A further temperature increase to 160 °C only gave **1** in 14% yield after 2 h due to the decomposition of the MOF (Figure S5), revealing that the combined effects of temperature and reaction media, such as base and solvent molecules, reduced the stability of the MOF. Considering the stability of Cu-TDPAT, all the subsequent experiments were conducted at 120 °C. In the absence of Cu-TDPAT, no product was obtained (Table 1, entry 1), illustrating that Cu-TDPAT promoted the *N*-arylation reaction.

**Table 1:** Goldberg-type C–N coupling reaction of iodobenzene and 5-methyl-2-(1*H*)-pyridone over copper MOFs and copper salts ^a^.

Entry	Catalyst	Solvent	Base	T (°C)	1 (%)	2 (%)
1	\	DMSO	K_2_CO_3_	120	n.r	n.r
2	Cu-TDPAT	DMSO	K_2_CO_3_	80	n.r	n.r
3	Cu-TDPAT	DMSO	K_2_CO_3_	100	9	1
4	Cu-TDPAT	DMSO	K_2_CO_3_	120	63 (90 ^b^)	3 (4 ^b^)
5	Cu-TDPAT	DMSO	K_2_CO_3_	140	70	3
6	Cu-TDPAT	DMSO	K_2_CO_3_	160	14	1
7	Cu-TDPAT	Toluene	K_2_CO_3_	120	n.r	n.r
8	Cu-TDPAT	1,2-Dichlorobenzene	K_2_CO_3_	120	n.r	n.r
9	Cu-TDPAT	DMF	K_2_CO_3_	120	34	2
10	Cu-TDPAT	1,4-Dioxane	K_2_CO_3_	120	n.r	n.r
11	Cu-TDPAT	DMSO	KOH	120	61	3
12	Cu-TDPAT	DMSO	Cs_2_CO_3_	120	57	3
13	Cu-TDPAT	DMSO	Et_3_N	120	n.r	n.r
14	Cu-TDPAT	DMSO	NaOMe	120	49	4
15	CuI	DMSO	K_2_CO_3_	120	80	4
16	CuCl	DMSO	K_2_CO_3_	120	71	5
17	Cu(OAc)_2_	DMSO	K_2_CO_3_	120	38	4
18	Cu(NO_3_)_2_	DMSO	K_2_CO_3_	120	29	2
19	CuBTC	DMSO	K_2_CO_3_	120	88	5
20	Cu(NO_3_)_2_ + BTC	DMSO	K_2_CO_3_	120	43	2
21	Cu(NO_3_)_2_ + TDPAT	DMSO	K_2_CO_3_	120	31	2

^a^ Reaction conditions: PhI (1 mmol), pyridone (1 mmol), MOFs (0.05 mmol, based on copper), base (2 mmol), solvent (5 mL), 2 h; ^b^ Data of reaction for 8 h; The symbol n.r represents no reaction.

It has been found that the choice of solvent is crucial to the outcome of C–N coupling reactions [16,29]. In order to determine the best reaction medium, the coupling reaction was also conducted in other solvents (Table 1, entries 7–10). DMSO was found to be the best solvent for the *N*-arylation of 5-methyl-2-(1*H*)-pyridone (Table 1, entry 4). Reaction in DMF gave the coupled product in a low yield. On the other hand, weakly polar solvents, like 1,2-dichlorobenzene and 1,4-dioxane, and non-polar solvents, such as toluene, were not suitable for this process, revealing the dramatic effects of the solvent on the C–N coupling reactions. Recent studies showed that DMSO acts as an oxidant for metal species in the formation of C–S and C–Se bonds by cross coupling reactions [79,80], but in our experiments, no dimethyl sulfide, the reduction product of DMSO, was detected, implying that here DMSO is only acting as an effective polar solvent. The effects of bases on the C–N coupling reaction was also investigated. Among the bases tested potassium carbonate, cesium carbonate, potassium hydroxide and sodium methoxide were effective for the formation of the desired product and gave similar results (Table 1, compare entries 4, 11, 12 and 14). On the other hand, triethylamine (Table 1, entry 13) did not effectively improve the *N*-arylation reaction, contrary to previously reported results [79]. One possibility is that the interaction of substrate molecules with catalyst was impeded due to the coordination of triethylamine with the copper ion on the MOF.

**Figure 2:**
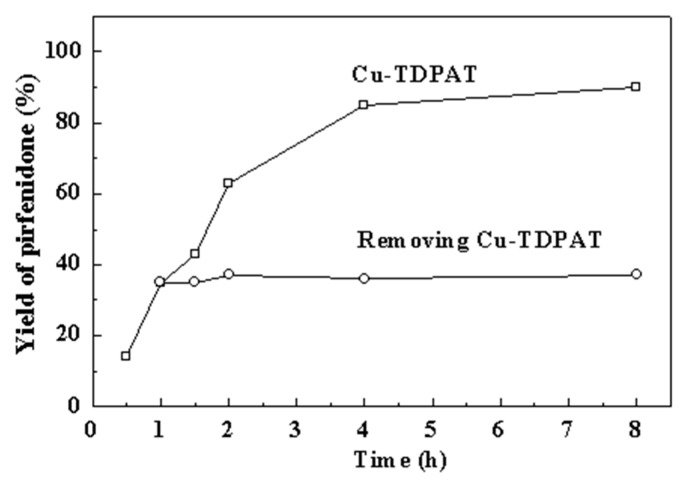
Yield as a function of reaction time in the coupling reaction of 5-methyl-2-(1*H*)-pyridone and iodobenzene with Cu-TDPAT as catalyst at 120 °C. □: catalyst present throughout; ○: catalyst removed from the suspension after 1 h. Reaction conditions: PhI (1 mmol), pyridone (1 mmol), Cu-TDPAT (0.05 mmol, based on copper), K_2_CO_3_ (2 mmol), DMSO (5 mL).

According to the above results, we conducted the C–N coupling reactions in DMSO at 120 °C for 8 h in the presence of K_2_CO_3_ as the standard reaction conditions. The coupling reaction afforded **1** as the main product in 90% yield and the by-product **2** in 4% yield, respectively (Figure 2, Table 1, entry 4). The existence of small amount of **2** reflected the fact that 5-methyl-2-(1*H*)-pyridone has an accessible tautomer, 5-methyl-2-hydroxypyridine, in the reaction system [81].

### 2.2. Heterogeneity of the Reaction

To verify whether the catalysis of Cu-TAPAT is truly heterogeneous or, on the contrary, is due to some leached copper species present in the reaction solutions, we performed a hot-filtration experiment: the Cu-TAPAT solid catalyst was removed from a hot solution by filtration one hour after initiating the catalytic test. The filtrate was further reacted for another 7 h. No significant catalytic conversion was observed (Figure 2), indicating that the reaction was terminated upon removal of the catalyst. On the other hand, inductively coupled plasma atomic emission spectroscopy (ICP-AES) analysis of the filtrate showed that there was only about 1 ppm of copper in the solution. These results suggested that the reaction proceeded over the MOF surface in a heterogeneous fashion, with the open copper sites on the axis of paddle-wheel SBUs within the MOF being responsible for the promotion of the C–N coupling reaction [82].

### 2.3. Comparison with Other Homogeneous Copper Catalysts

For comparison, several copper salts, CuI, CuCl, Cu(OAc)_2_ and Cu(NO_3_)_2_, were used as homogeneous catalysts for the same reaction (Table 1, entries 15–18). The experimental results showed that both copper(I) salts—CuI and CuCl—produced pirfenidone in high yield, while the copper(II) salts resulted in relative low yields of coupled product. A widely used MOF, namely CuBTC [83] (Figures S6 and S7) that was constructed from paddle-wheel type copper clusters and 1,3,5-benzenetricarboxylate molecules, also exhibited high activity in the C–N coupling reaction between iodobenzene and 5-methyl-2-(1*H*)-pyridone (Table 1, entry 19), but the poor stability of CuBTC under the reaction conditions prevented its recycling. Similar phenomena were reported by Garcia [75] and Kantam [84]. In the case of CuBTC, a high yield (88%) of **1** was obtained, which was much higher than that of the homogeneous catalytic processes catalyzed by Cu(NO_3_)_2_ or both Cu(NO_3_)_2_ and BTC (Table 1, entries 18 and 20). Moreover, the same result was also observed in the comparison of Cu-TDPAT and Cu(NO_3_)_2_/TDPAT (Table 1, entries 4 and 21). Owing to the same amount of copper species being used in the heterogeneous and homogeneous reaction systems, the above results further reveal that the high density of open copper sites within the MOF were the active sites of the MOF catalyst and responsible for the enhanced C–N coupling reaction rates.

### 2.4. Reusability of the Cu-TDPAT Catalyst

**Figure 3:**
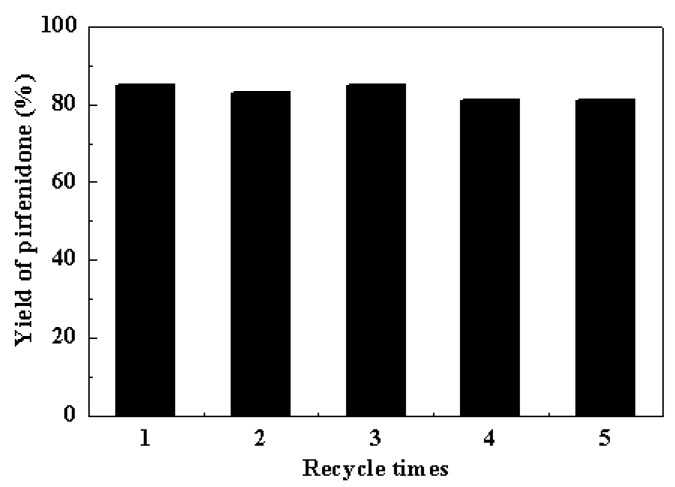
Reuse of Cu-TDPAT in the Goldberg-type C–N coupling reaction of iodobenzene and 5-methyl-2-(1*H*)-pyridone. Reaction condition: PhI (1 mmol), pyridone (1 mmol), Cu-TDPAT (0.05 mmol, based on copper), K_2_CO_3_ (2 mmol), DMSO (5 mL), 4 h, temperature 120 °C.

A marked advantage of heterogeneous catalysis is the possibility of recovering and reusing the catalysts after the reaction. The recycle of the Cu-TDPAT catalyst was further examined in the coupling reaction of 5-methyl-2-(1*H*)-pyridone with iodobenzene (Figure 3). Pirfenidone was obtained in 85%, 83%, 85%, 81% and 81% yields in successive 4 h cycles. The results demonstrated that Cu-TDPAT exhibited good reusability in the Goldberg-type C–N coupling reaction. The image (Figure S1b) and XRD patterns (Figure 1) of the Cu-TDPAT catalyst after five reaction runs indicated that the particle shape, the crystallinity and the chemical structure of Cu-TDPAT were almost completely maintained. The presence of a little bit of powder can be attributed to the degradation of a few crystal particles induced by the stirring and the effect of the reaction medium. The nitrogen physisorption measurements further demonstrated the porosity and stability of Cu-TDPAT after use (Figure S3). The changes in surface area and pore volume of the used Cu-TDPAT (Table S1) are probably due to the adsorption of a few reactant or product molecules in the MOF cavities [85,86]. This hypothesis was supported by the changes of thermogravimetric data, in which more weight loss (about 2%) was observed for the used Cu-TDPAT sample (Figure S4).

### 2.5. Generality of the N-Arylation Reaction

The generality of the C–N coupling reaction was investigated under the standard reaction conditions. Initially various halobenzenes were subjected to the reaction conditions. It was found that bromobenzene provided the corresponding *N*-arylated pyridone (Table 2, entry 1) and *N*-arylated pyrrolidone (Table 2, entry 2) derivatives in moderate yield, while chlorobenzene was unreactive under these conditions (Table 2, entry 3). Then, the *N*-arylation reactions of other primary and secondary amines with iodobenzene were examined (Table 2, entries 4–12). The results showed that a wide range of amines, including aliphatic amines, aryl amines and N–H heterocycles, could undergo the C–N coupling reactions smoothly to afford the expected *N*-arylation products in moderate yields. 

**Table 2:** Ullmann and Goldberg type C–N coupling reactions of halobenzenes with various amines and amides over Cu-TDPAT *.

Entry	(R_1_R_2_)NH	ArX	Yield (%)
1	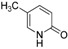	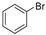	39 (2 ^a^)
2	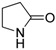	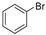	37
3	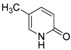	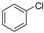	n.r
4	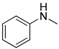	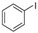	51
5	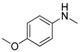	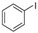	54
6	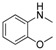	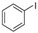	43
7	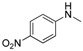	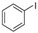	35
8	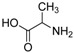	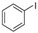	59
9	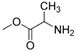	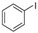	55
10	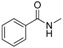	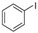	56
11	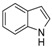	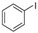	72 (93 ^b^)
12	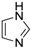	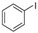	61 (91 ^b^)

* Reaction conditions: halobenzenes (1 mmol), amine derivatives (1 mmol), MOFs (0.05 mmol, based on copper), K_2_CO_3_ (2 mmol), DMSO (5 mL), 2 h, temperature 120 °C. ^a^ The yield of **2**; ^b^ Data of reaction for 8 h. The symbol of n.r represents no reaction.

In the case of indole, the coupling product, 1-phenylindole, can be obtained in 93% yield after 8 h, which is comparable to the homogeneous system results [28,79], indicating the high activity of Cu-TDPAT. *N*-Methylaniline derivatives bearing electron-withdrawing and electron-donating groups on the benzene ring afforded obviously different results under the standard reaction conditions. When 4-methoxy-*N*-methylaniline was subjected to the reaction, a good yield (54%) of the desired product was obtained thanks to the electron-donating effect of the methoxy group. However the coupling reactions of 4-nitro-*N*-methylaniline proceeded with difficulty to give the *N*-arylation product in a low yield (Table 2, entry 7). These results demonstrated that the electronic properties of the substituent(s) on the aryl amine play an important role in determining its reactivity in the C–N coupling reactions. Compared to 4-methoxy-*N*-methylaniline, the reaction of 2-methoxy-*N*-methylaniline gave a relative low yield of the expected product due to the steric hindrance of the adjacent methoxy group (Table 2, entry 6). Secondary amides are a particularly challenging substrate class for cross couplings due to the large size of secondary amides and their relatively low nucleophilicity (compared to amines) [87,88]. The reaction of *N*-methylbenzamide (Table 2, entry 10) with iodobenzene afforded a 56% yield of the tertiary amide, *N*-methyl-*N*-phenylbenzamide, within 2 h in the *N*-arylation reaction, implying that Cu-TDPAT showed a higher activity than copper(I) thiophenecarboxylate (CuTC) [88] in the *N*-arylation reaction of secondary amides.

Table 3 provides a comparison of the results obtained for the Cu-TDPAT catalytic system with other reported heterogeneous catalytic systems in the cross-coupling reaction of imidazole with iodobenzene, further indicating the higher activity of this Cu(II) coordination polymer compared to the other reported systems. Considering the chemical structure and *rht*-topology of Cu-TDPAT, its high catalytic activity can be attributed to the high density of open copper sites within Cu-TDPAT framework and the high pore volume and large open windows that may reduce the diffusional limitation of the substrate and product [85].

**Table 3:** Comparison of activity of different heterogeneous catalysts in the *N*-arylation reaction of imidazole and iodobenzene.

Catalyst *	Reaction Conditions	Yield	Reference
CuI/Meso-*N*-*C*-1	DMSO, KOH, 125 °C, 24 h	88%	[42]
CuI/PSP	H_2_O, K_3_PO_4_, 120 °C, 8 h	65%	[48]
Cu-MPTA-1	H_2_O, KOH, 120 °C, 12 h	91%	[52]
Cu/SiO_2_	Toluene, Cs_2_CO_3_, 100 °C, 8 h	23%–92%	[44]
Cu-TDPAT	DMSO, K_2_CO_3_, 120 °C, 8 h	91%	This study

* Meso-*N*-*C*-1, PSP, MPTA-1 and SiO_2_ represent mesoporous nitrogen doped carbon, polystyrene-supported pyrrole-2-carbohydrazide, mesoporous polytriallylamine, and amine or imine-modified silica, respectively.

### 2.6. Mechanistic Considerations

Although the mechanism of Ullmann and Goldberg type C–N coupling reactions in the presence of copper compounds has been widely studied, and four mechanistic pathways involving oxidative addition/reductive elimination, single electron transfer (SET), σ-bond metathesis and π-complexation, respectively, have been proposed [89,90], copper-mediated C–N coupling reactions are still in some sense unpredictable. In order to investigate the function of Cu-TDPAT in the coupling reaction, X-band (9.06 GHz) electron paramagnetic resonance (EPR) spectra of the reaction system were recorded at 90 °C. The EPR spectra of the reaction mixture were dominated by a broad signal at g = 2.107 (Figure S8), which could be attributed to Cu^2+^–Cu^2+^ dimers or mononuclear Cu^2+^ ions [91,92], indicating that the copper atoms on the MOF catalyst exist mainly as Cu(II) species in the reaction mixtures. Compared to the spectra of pure Cu-TDPAT (Figure S9), the decrease of the intensity of EPR signal of the reaction mixture with time and the change of g-value indicated the occurrence of interaction between reaction substrates (iodobenzene and 5-methyl-2-(1*H*)-pyridone) and copper sites. No any organic radical species was detected, revealing that the SET mechanism could be discounted. This was further supported by the experiment in which no effect on the turnover frequency of the coupling reaction was observed when the free radical scavenger 2,2,6,6-tetramethyl-1-piperidinyloxy (TEMPO) was added to the reaction system. Moreover, the σ-bond metathesis pathway and oxidative addition/reductive elimination pathway could also be eliminated due to the difficulty in forming the corresponding intermediates, because the halobenzene molecules and amine species bonding to the same copper ion from axis might dissociate the MOF, rendering it non-recyclable. Therefore a possible mechanism for the *N*-arylation is that the halobenzene molecule interacts with copper ion first and forms a π-complex, then the amine species attacks the halobenzene activated by copper, giving the coupling product.

## 3. Experimental Section

### 3.1. General Information

All solvents and chemicals were obtained commercially and were used as received without further purification. Cu-TDPAT [76] and CuBTC [83] were synthesized according to the reported procedures, respectively. The catalysts samples were activated as follows: the as-synthesized MOF sample was soaked in dry methanol for 12 h, separated from the mixture, and then the process was repeated four times to remove the high boiling point solvates used in preparations.

Powder X-ray diffraction was performed on a D8 Advance instrument (Bruker, Karlsruhe, Germany) using Cu–Kα radiation (λ = 1.5406 Å) at room temperature with a scan speed of 0.5 s per step and a step size of 0.02°. ^1^H-NMR and ^13^C-NMR data were collected on Bruker ARX-400 or Bruker ARX-600 spectrometers (Bruker) at 400 MHz and 101 or 151 MHz, respectively, using CDCl_3_ or DMSO-*d*_6_ solutions with tetramethylsilane as an internal standard. Molecular weights were obtained on an Aglilent 6410 Triple Quad LC-MS mass spectrometer (Agilent Technologies, Palo Alto, CA, USA). Scanning electron microscope (SEM) images were obtained on Hitachi S 4300 (Hitachi, Tokyo, Japan) and JEOL-2010 (JEOL, Tokyo, Japan) instruments at room temperature. Inductively coupled plasma atomic emission spectroscopy (ICP-AES) analysis was performed on an IRIS Intrepid ER/S instrument (Thermo Elemental, Waltham, MA, USA). Nitrogen sorption measurement was performed at −196 °C on a ASAP 2020 (Micromeritics, Norcross, GA, USA). A sample of approximately 50 mg was outgassed at 150 °C for 12 h and then nitrogen isotherm at −196 °C was measured in liquid nitrogen bath using UHP-grade (99.999%) gas source. The Brunauer-Emmett-Teller (BET) surface area of Cu-TDPAT and CuBTC was calculated based on the nitrogen absorption isotherm. Thermogravimetric analysis (TGA) was performed on a TG 209F3 instrument (Netzsch, Selb, Germany) under nitrogen atmosphere (250 mL/min). The sample was heated at a constant rate of 10 °C/min from 40 °C to 400 °C. The continuous wave (CW) EPR spectra of the reaction mixture in toluene at X-Band were measured using a JES-FA200 spectrometer (JEOL), microwave frequency 9.06 GHz, power of the microwave 0.998 mW) at 90 °C. The data for pure Cu-TDPAT were obtained from powder samples at room temperature.

### 3.2. Catalytic Reactions

The coupling reaction was performed as follows: in a typical process, 5-methyl-2-(1*H*)-pyridone (1 mmol), aryl halide (1 mmol), base (2 mmol) and solvent (5 mL) were added to an oven-dried tube containing 5% (based on copper) MOF catalyst or copper salt. The mixture was stirred at desired temperature for 2 h. After being cooled to room temperature, the catalyst was filtrated and washed with ethyl acetate. The products were isolated by a series 1500 preparative high performance liquid chromatography system (SSI, Charlotte, NC, USA) equipped with a UV-VIS detector, using a Kromasil C18 column (50 × 250 mm) and gradient elution with a H_2_O (A)-acetonitrile (B) the mobile phase. The gradient program was 0 min, 10% B; 20 min, 35% B. The flow rate of mobile phase was 40 mL/min, and the detection wavelength was 220 nm. Fractions were collected and evaporated to afford the pure products.

*5-Methyl-1-phenyl-2-(1H)-pyridone*. ^1^H-NMR (400 MHz; CDCl_3_): δ 7.41 (t, *J* = 7.0 Hz, 2H), 7.36 (t, *J* = 7.7 Hz, 1H), 7.32 (d, *J* = 7.8 Hz, 2H), 7.21 (d, *J* = 9.2 Hz, 1H), 7.07 (s, 1H), 6.53 (d, *J* = 9.2 Hz, 1H), 2.04 (s, 3H); ^13^C-NMR (151 MHz; DMSO-*d*_6_): δ 160.95, 143.52, 141.47, 136.49, 129.46, 128.42, 127.17, 120.67, 114.60, 16.77; MS (ESI) *m*/*z*: 186.23 ([M + H]^+^; Calcd for C_12_H_11_NO + H 186.09, found: 186.23).

*5-Methyl-2-phenoxypyridine*. ^1^H-NMR (400 MHz, DMSO-*d*_6_): δ 7.98 (d, *J* = 1.7 Hz, 1H), 7.66 (dd, *J* = 8.3 Hz, 2.3 Hz, 1H), 7.39 (t, *J* = 7.9 Hz, 2H), 7.17 (t, *J* = 7.4 Hz, 1H), 7.07 (d, *J* = 7.8 Hz, 2H), 6.92 (d, *J* = 8.3 Hz, 1H), 2.23 (s, 3H). ^13^C-NMR (151 MHz; DMSO-*d*_6_): δ 161.63, 154.89, 147.41, 141.16, 130.08, 128.52, 124.57, 121.15, 111.62, 17.38. MS (ESI) *m*/*z*: 186.23 ([M + H]^+^; Calcd for C_12_H_11_NO + H 186.09, found: 186.23).

*N-Methyldiphenylamine*. ^1^H-NMR (400 MHz, CDCl_3_): δ 7.40 (t, *J* = 7.9Hz, 4H), 7.15 (d, *J* = 8.2 Hz, 4H), 7.08 (t, *J* = 7.3 Hz, 2H), 3.43 (s, 3H). ^13^C-NMR (151 MHz; DMSO-*d*_6_): δ 149.09, 129.62, 121.57, 120.57, 40.01. MS (ESI) *m*/*z*: 184.11 ([M + H]^+^; Calcd for C_13_H_13_N + H 184.11, found: 184.11).

*N-Methyl-N-(4-methoxy)phenylaniline*. ^1^H-NMR (400 MHz, CDCl_3_): δ 7.24–7.17 (m, 2H), 7.17–7.12 (m, 2H), 7.00 (m, 1H), 6.84–6.77 (m, 2H), 6.69–6.64 (m, 2H), 3.88 (s, 3H), 3.31 (s, 3H). ^13^C-NMR (151 MHz; DMSO-*d*_6_): δ 156.36, 149.92, 142.13, 129.30, 126.35, 118.55, 115.89, 115.27, 55.65, 40.63. MS (ESI) *m*/*z*: 214.09 ([M + H]^+^; Calcd for C_14_H_15_NO + H 214.12, found: 214.09).

*N-Methyl-N-(2-methoxy)phenylaniline*. ^1^H-NMR (400 MHz, DMSO-*d*_6_): δ 7.33–7.22 (m, 1H), 7.17 (dd, *J* = 12.3 Hz, 3 Hz, 1H), 7.13 (d, *J* = 6.1 Hz, 1H), 7.11 (t, *J* = 7.9 Hz, 2H), 6.99 (t, *J* = 7.5 Hz, 1H), 6.62 (t, *J* = 7.2 Hz, 1H), 6.51 (d, *J* = 8.2 Hz, 2H), 3.71 (s, 3H), 3.13 (s, 3H). ^13^C-NMR (151 MHz; DMSO-*d*_6_): δ 152.13, 148.33, 142.22, 129.12, 123.74, 122.84, 121.88, 119.22, 119.13, 113.32, 56.79, 37.87. MS (ESI) *m*/*z*: 214.09 ([M + H]^+^; Calcd for C_14_H_15_NO + H 214.12, found: 214.09).

*N-Methyl-N-(4-nitro)phenylaniline*. ^1^H-NMR (400 MHz, DMSO-*d*_6_): δ 8.05 (d, *J* = 6.2 Hz, 2H), 7.50 (t, *J* = 7.8 Hz, 2H), 7.35 (t, *J* = 7.5 Hz, 1H), 7.31 (d, *J* = 6.7 Hz, 2H), 6.74 (d, *J* = 6.3 Hz, 2H), 3.38 (s, 3H). ^13^C-NMR (151 MHz; DMSO-*d*_6_): δ 154.13, 146.44, 137.58, 130.67, 127.16, 126.96, 126.10, 113.02, 40.85. MS (ESI) *m*/*z*: 229.11 ([M + H]^+^; Calcd for C_13_H_12_N_2_O_2_ + H 229.10, found: 229.11).

*2-(Phenylamino)propanoic acid*. ^1^H-NMR (400 MHz, DMSO-*d*_6_): δ 7.06 (t, *J* = 7.8 Hz, 2H), 6.55 (d, *J* = 8 Hz, 2H), 6.55–6.54 (m, 1H), 3.92 (q, *J* = 7.0 Hz, 1H), 1.36 (d, *J* = 7.0 Hz, 3H). ^13^C-NMR (101 MHz; DMSO-*d*_6_): δ 177.62, 147.78, 128.83, 116.25, 112.46, 51.32, 18.21. MS (ESI) *m*/*z*: 166.09 ([M + H]^+^; Calcd for C_9_H_11_NO_2_ + H 166.09, found: 166.09).

*Methyl 2-(phenylamino)propanoate*. ^1^H-NMR (400 MHz, DMSO-*d*_6_): δ 7.07 (t, *J* = 7.8 Hz, 2H), 6.58–6.56 (m, 1H), 6.53 (d, *J* = 7.7 Hz, 2H), 4.05 (q, *J* = 7.0 Hz, 1H), 3.62 (s, 3H), 1.37 (d, *J* = 7.0 Hz, 3H). ^13^C-NMR (151 MHz, DMSO-*d*_6_): δ 174.96, 147.57, 128.90, 116.45, 112.30, 50.90, 50.70, 18.12 (s, 1H). MS (ESI) *m*/*z*: 180.11 ([M + H]^+^; Calcd for C_10_H_13_NO_2_ + H 180.10, found: 180.11).

*N-Phenyl-2-pyrrolidone*. ^1^H-NMR (400 MHz, DMSO-*d*_6_): δ 7.65 (d, *J* = 7.8 Hz, 2H), 7.37 (t, *J* = 8.0 Hz, 2H), 7.12 (t, *J* = 7.4 Hz, 1H), 3.83 (t, *J* = 7.0 Hz, 2H), 2.48 (t, *J* = 8.4 Hz, 2H), 2.06 (m, *J* = 7.5 Hz, 2H). ^13^C-NMR (151 MHz; DMSO-*d*_6_): δ 174.28, 140.07, 129.06, 124.26, 119.80, 48.50, 32.78, 17.86. MS (ESI) *m*/*z*: 162.09 ([M + H]^+^; Calcd for C_10_H_11_NO + H 162.09, found: 162.09).

*N-Methyl-N-benzoylaniline*. ^1^H-NMR (400 MHz, CDCl_3_): δ 7.30 (t, *J* = 7.2 Hz, 2H), 7.21 (t, *J* = 7.4 Hz, 2H), 7.18 (t, *J* = 4.5 Hz, 1H), 7.14 (d, *J* = 8.1 Hz, 2H), 7.11 (t, *J* = 6.4 Hz, 1H), 7.02 (d, *J* = 7.5 Hz, 2H), 3.48 (s, 3H). ^13^C-NMR (151 MHz; DMSO-*d*_6_): δ 170.01, 145.02, 136.77, 129.82, 129.53, 128.64, 128.19, 127.50, 126.88, 38.34. MS (ESI) *m*/*z*: 212.11 ([M + H]^+^; Calcd for C_14_H_13_NO + H 212.11, found: 212.11).

*N-Phenylindole*. ^1^H-NMR (400 MHz, DMSO-*d*_6_): δ 7.68 (d, *J* = 7.6 Hz, 1H), 7.64 (d, *J* = 3.3 Hz, 1H), 7.59–7.54 (m, 5H), 7.45–7.35 (m, 1H), 7.09–7.24 (m, 2H), 6.75 (d, 1H). ^13^C-NMR (151 MHz; DMSO-*d*_6_): δ 139.63, 135.62, 130.26, 129.65, 128.84, 126.82, 124.24, 122.80, 121.48, 120.76, 110.82, 104.06. MS (ESI) *m*/*z*: 194.11 ([M + H]^+^; Calcd for C_14_H_11_N + H 194.10, found: 194.11).

*N-Phenylimidazole*. ^1^H-NMR (400 MHz, DMSO-*d*_6_): δ 8.32 (br, 1H), 7.79 (br, 1H), 7.66 (d, *J* = 7.6 Hz, 2H), 7.52 (t, *J* = 7.8 Hz, 2H), 7.43–7.29 (m, 1H), 7.17 (br, 1H). ^13^C-NMR (101 MHz; DMSO-*d*_6_): δ 136.94, 135.60, 129.93, 129.84, 126.92, 120.40, 118.30. MS (ESI) *m*/*z*: 145.08 ([M + H]^+^; Calcd for C_9_H_8_N_2_ + H 145.08, found: 145.08).

### 3.3. Reuse of Cu-TDPAT

The aforementioned procedure was used with 5-methyl-2-(1*H*)-pyridone (1 mmol), iodobenzene (1 mmol), K_2_CO_3_ (2 mmol), DMSO (5 mL) and 5% Cu-TDPAT (based on copper). The reaction mixture was magnetically stirred at 120 °C for 4 h. The liquid solution was removed, and the solid mixture was washed with ethyl acetate (5 mL). The resulting solid phase was reused for further reactions without previous purification.

## 4. Conclusions

In summary, a detailed investigation of *N*-arylation reaction of 5-methyl-2-(1*H*)-pyridone with halobenzenes was carried out using Cu-TDPAT, a metal-organic framework with high density of open copper sites within its framework, as an efficient heterogeneous catalyst. It has been demonstrated that Cu-TDPAT can improve the *N*-arylation reaction of a wide variety of amines and amides with iodobenzene or bromobenzene efficiently. The experimental results prove that Cu-TDPAT is stable to the conditions of Ullmann and Goldberg type coupling reactions, and the open copper sites on the axis of paddle-wheel SBUs within MOF are responsible for the promotion of the C–N coupling reaction. Further work is in progress to broaden the scope of this catalytic system to other substrates and to better understand the reaction mechanism.

## Supplementary Materials

Supplementary materials can be accessed at: http://www.mdpi.com/1420-3049/20/12/19756/s1.
