# Supplementary material for: A Copper-Based Metal-Organic Framework as an Efficient and Reusable Heterogeneous Catalyst for Ullmann and Goldberg Type C–N Coupling Reactions
Source: Molecules. 2015 Nov 27;20(12):21178–92. doi: 10.3390/molecules201219756 (PMC6332410; doi:10.3390/molecules201219756)
Supplement: Supplementary file 1 [file molecules-20-19756-s001.pdf]

Supplementary Materials: A Copper Based Metal-Organic Framework as an Efficient and Reusable Heterogeneous Catalyst for Ullmann and Goldberg Type C–N Coupling Reactions

Wei Long, Wenge Qiu, Chongwei Guo, Chuanqiang Li, Liyun Song, Guangmei Bai, Guizhen Zhang and Hong He

1. Additional Figures

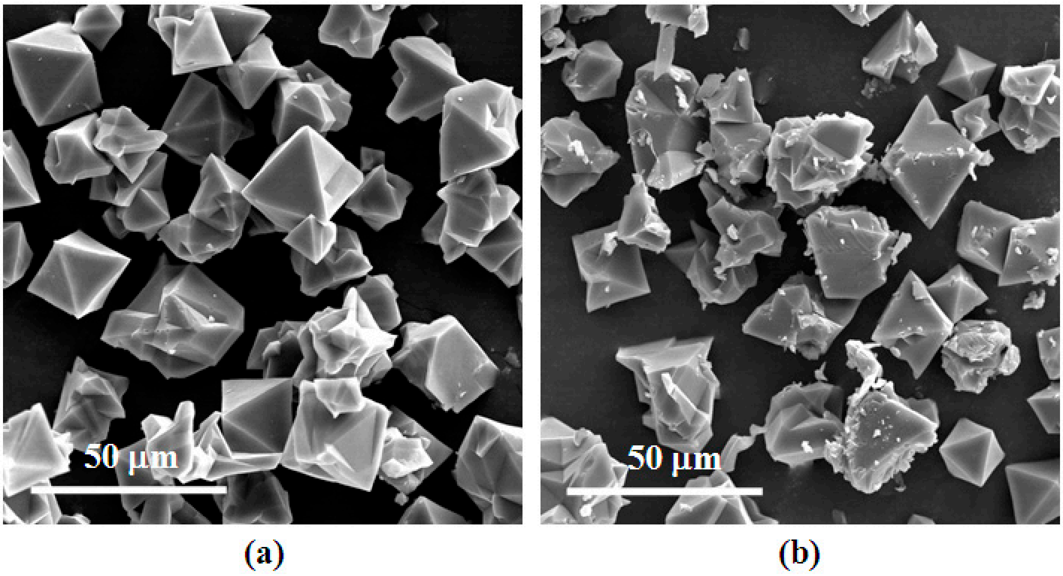

Figure S1. SEM images of fresh (**a**); and used after five catalysis cycles (**b**) Cu-TDPAT samples.

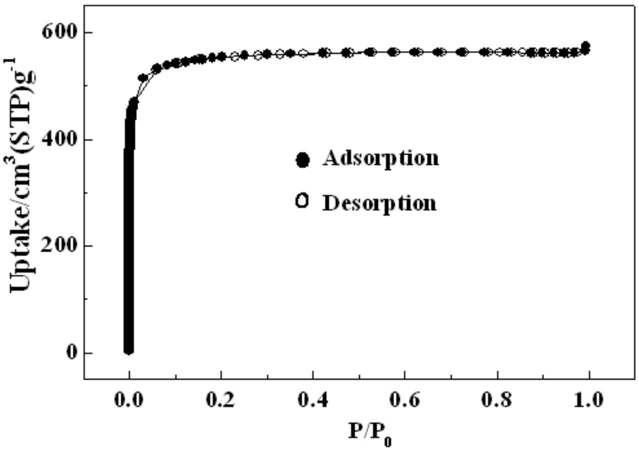

Figure S2. Nitrogen sorption isotherms of fresh Cu-TDPAT sample at 77 K.

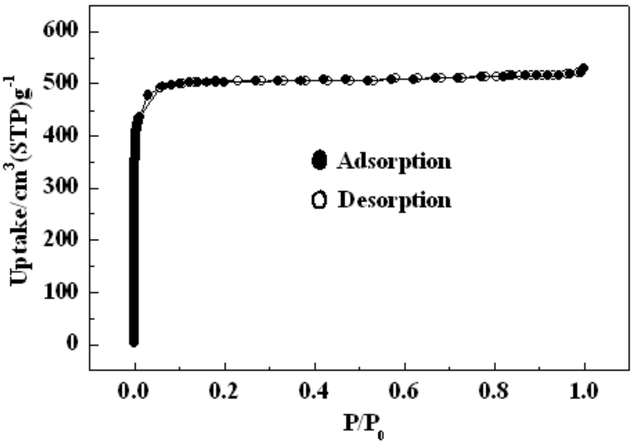

Figure S3. Nitrogen sorption isotherms of used Cu-TDPAT sample after five catalysis cycles at 77 K.

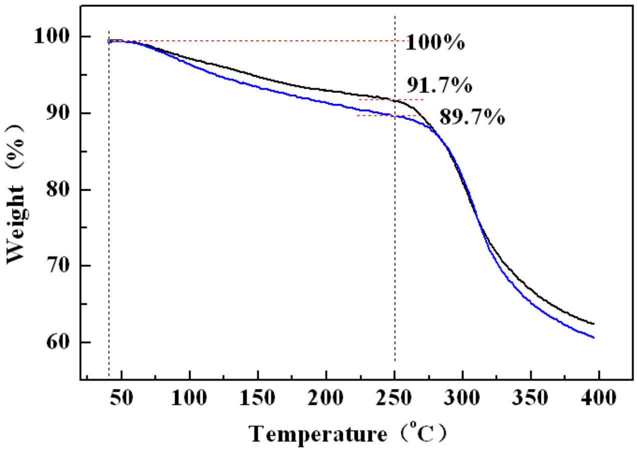

Figure S4. TGA data for the fresh Cu-TDPAT and used Cu-TDPAT samples after five catalysis cycles. The continuous weight loss of 8.3% from room temperature to 250 °C for the fresh Cu-TDPAT sample or the used Cu-TDPAT sample could be attributed to the loss of physisorbed and coordinated water and solvent molecules. Considering the samples were pretreated under the same conditions, the increased weight loss (about 2%) of the used Cu-TDPAT sample was attributed to the adsorption of a few reactant or product molecules in the MOF cavities.

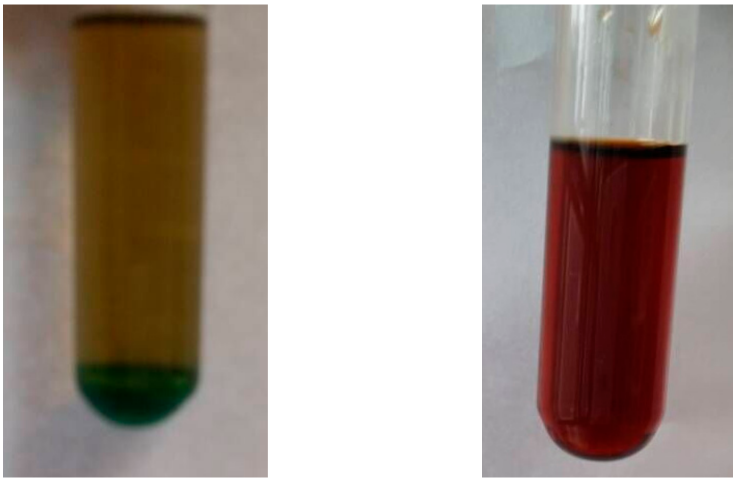

(**a**) (**b**)

Figure S5. Images of the reaction mixtures after 2 h at 120 °C (**a**); and at 160 °C (**b**), respectively. The disappearance of green powder on the bottom indicated the breakage of the MOF framework.

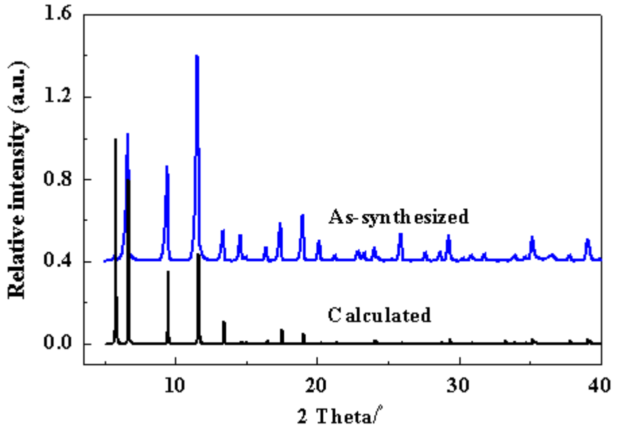

Figure S6. X-ray diffraction patterns for the as-synthesized CuBTC sample.

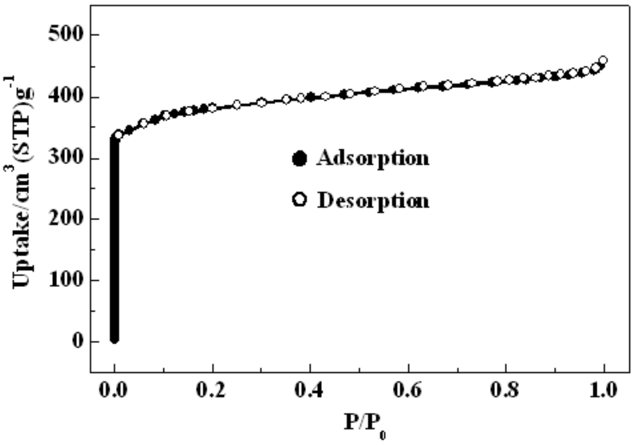

Figure S7. Nitrogen sorption isotherms of fresh CuBTC sample (The BET surface area of CuBTC was 1295 m^2/g).

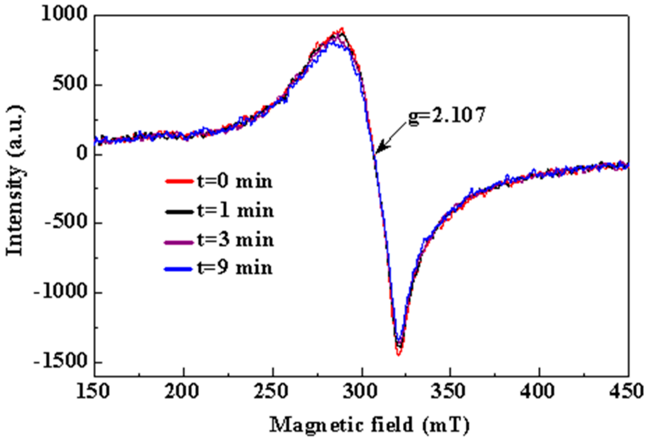

Figure S8. Continuous wave (CW) EPR spectra of the reaction mixture in toluene at X-band at 90 °C. The decrease of intensity of the EPR signal of the reaction mixture from 0 min to 9 min was about 3.7%, then it became stable.

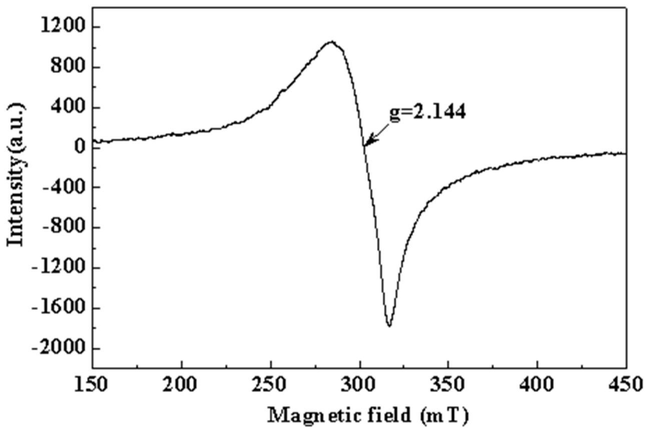

Figure S9. Continuous wave (CW) EPR spectra of Cu-TDPAT at X-band at room temperature.

Table S1. Characterization results of fresh and used five catalysis cycles Cu-TDPAT samples.

	S_{BET} (m^2/g)	$V_{total}/cm^3 \cdot g^{-1}$	$D_{average}/nm$
Fresh sample	1855	0.889	1.92
Used sample	1680	0.773	1.84

2. ¹H-NMR Spectra of All the Products

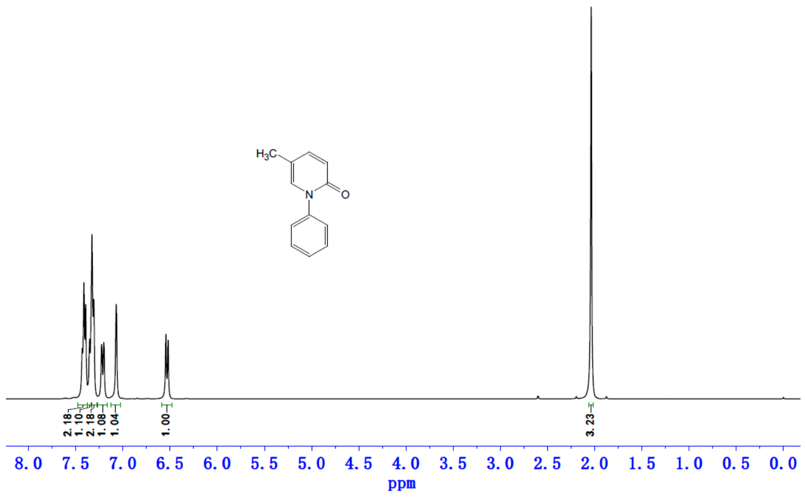

5-Methyl-1-phenyl-2-(1H)-pyridone

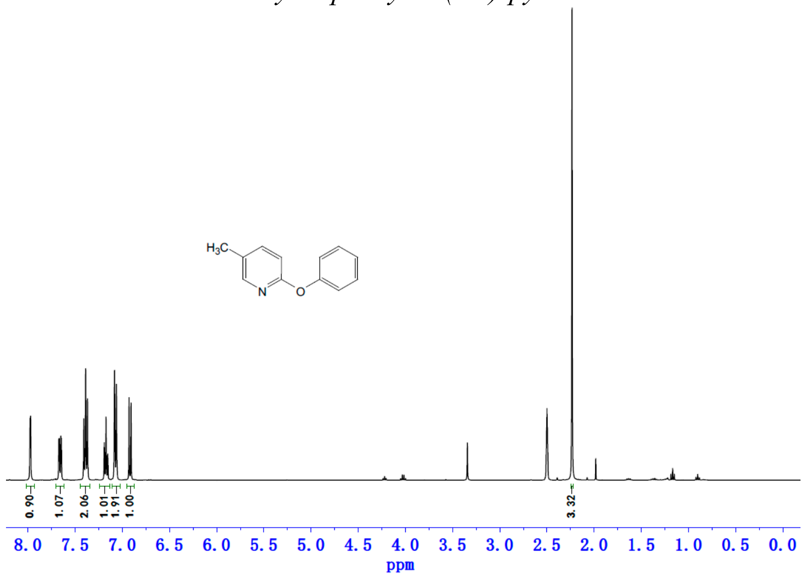

5-Methyl-2-phenoxypyridine

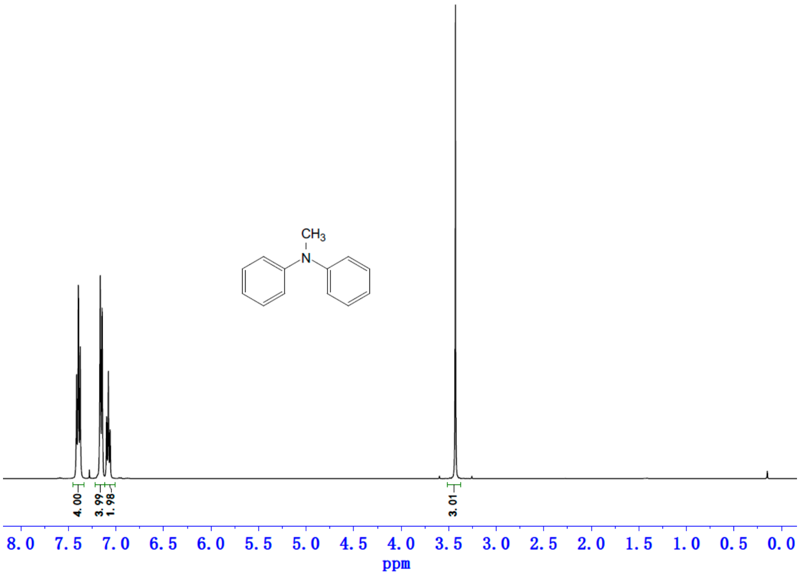

N-Methyldiphenylamine

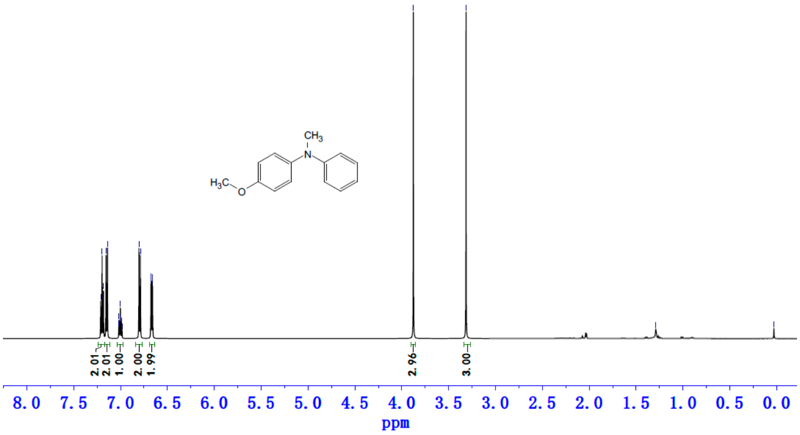

N-Methyl-N-(4-methoxy)phenylaniline

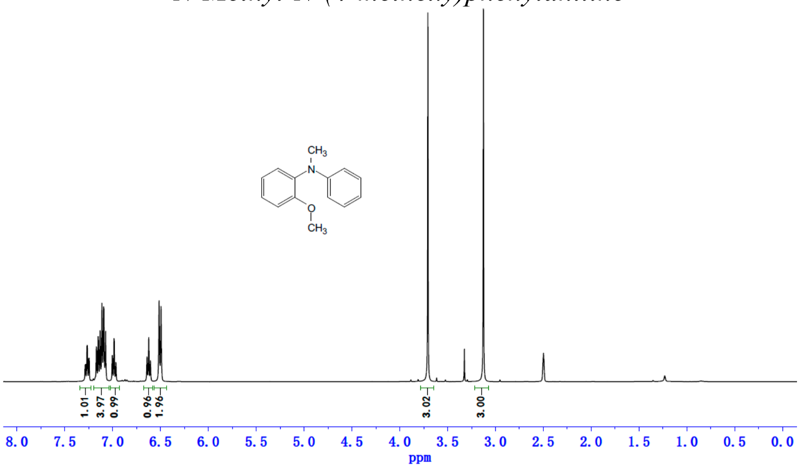

N-Methyl-N-(2-methoxy)phenylaniline

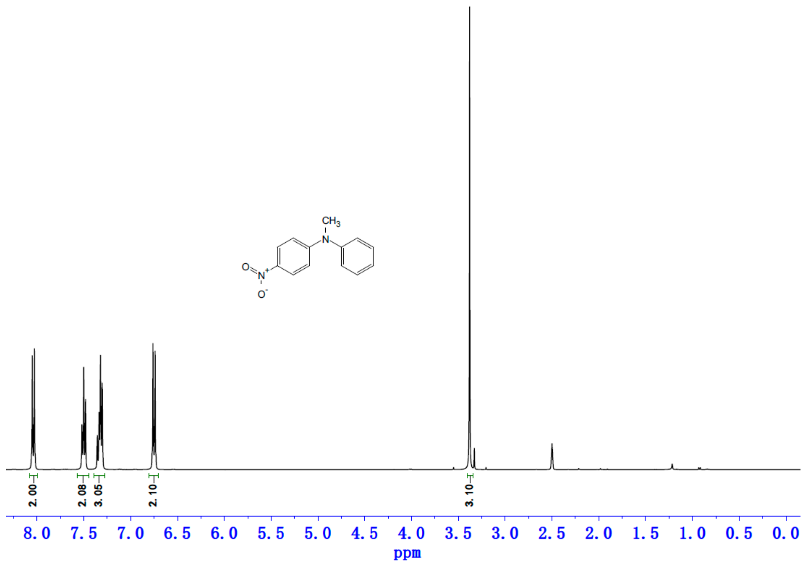

N-Methyl-N-(4-nitro)phenylaniline

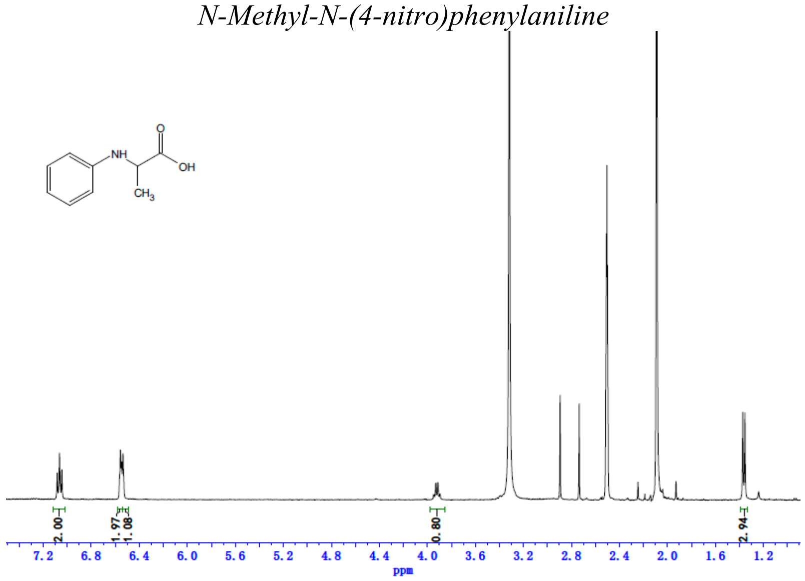

2-(Phenylamino)propanoic acid

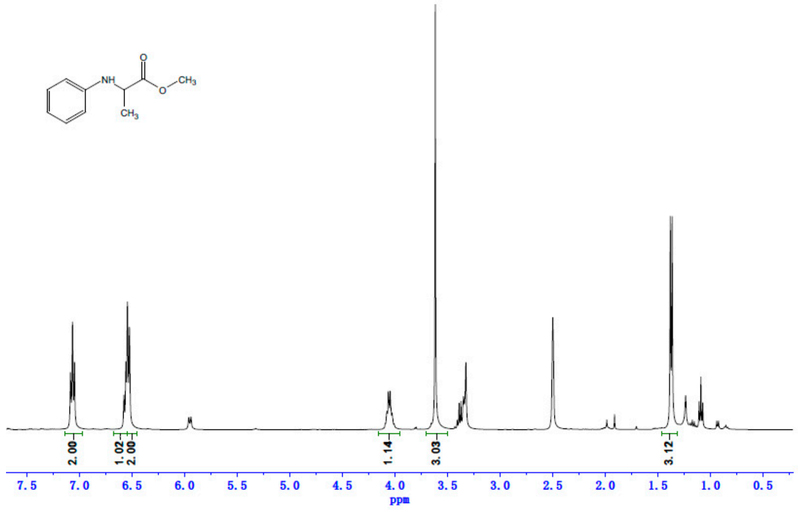

Methyl 2-(phenylamino)propanoate

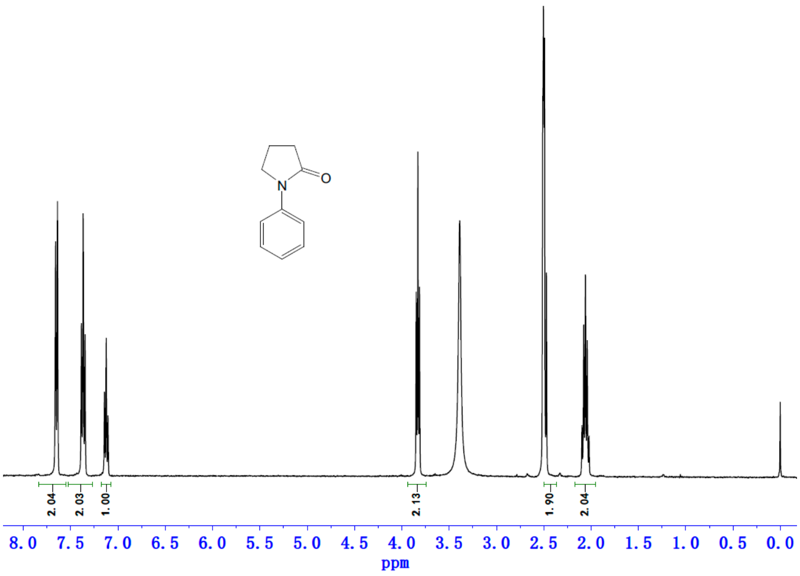

N-Phenyl-2-pyrrolidone

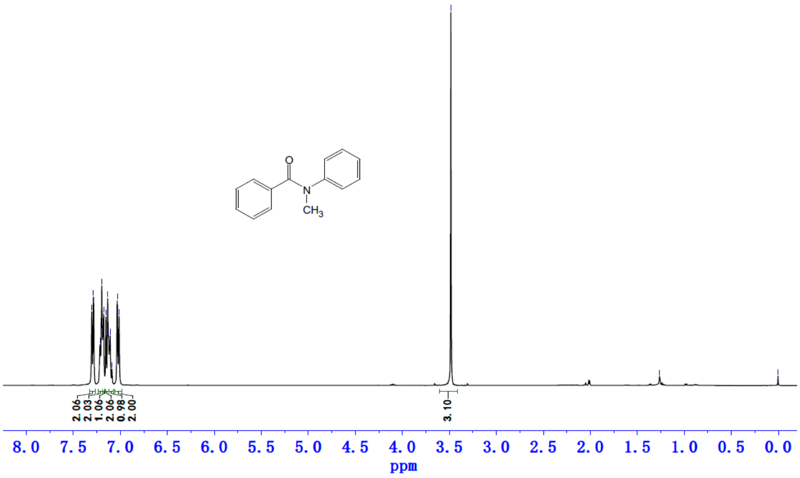

N-Methyl-N-benzoylaniline

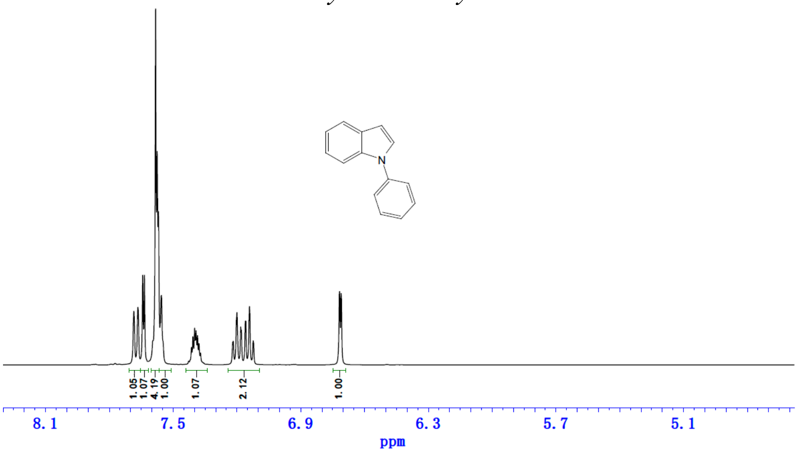

N-Phenylindole

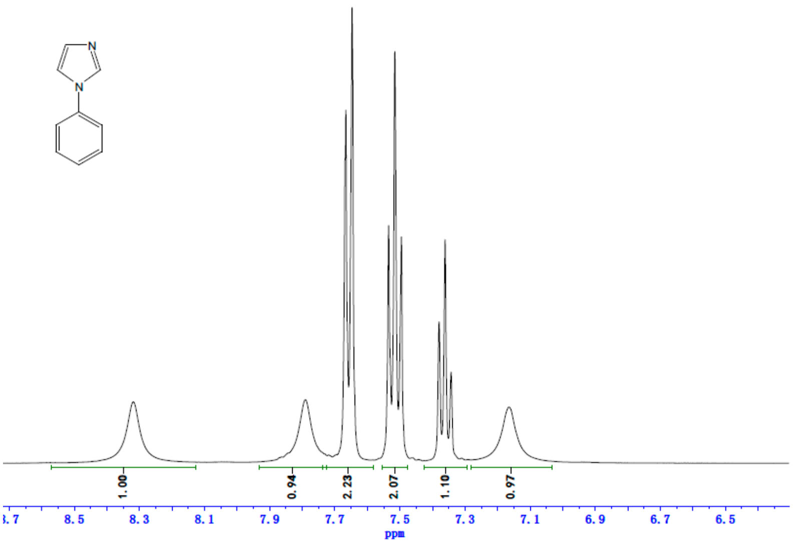

N-Phenylimidazole

3. ^{13}C-NMR Spectra of All the Products

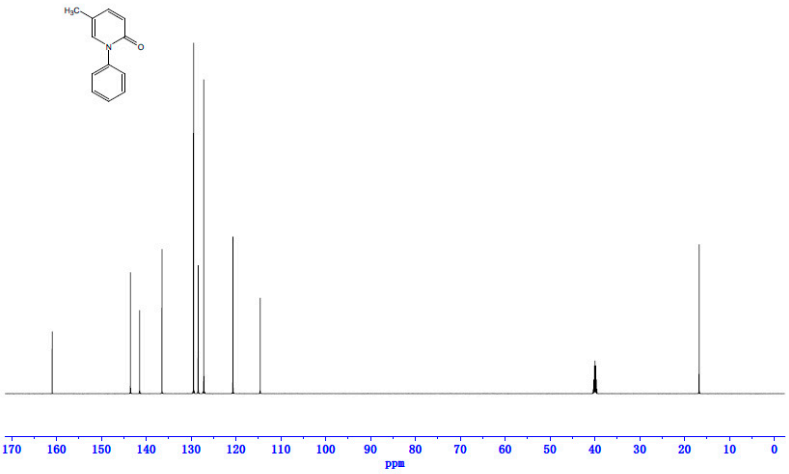

5-Methyl-1-phenyl-2-(1H)-pyridone

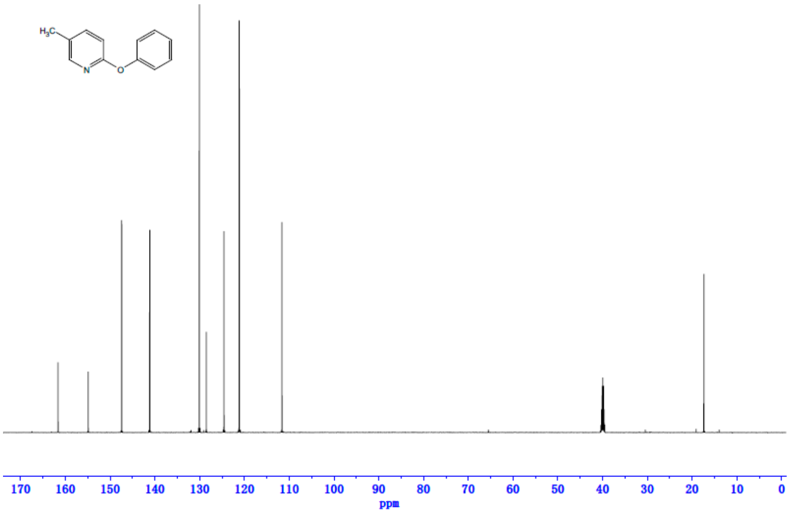

5-Methyl-2-phenoxypyridine

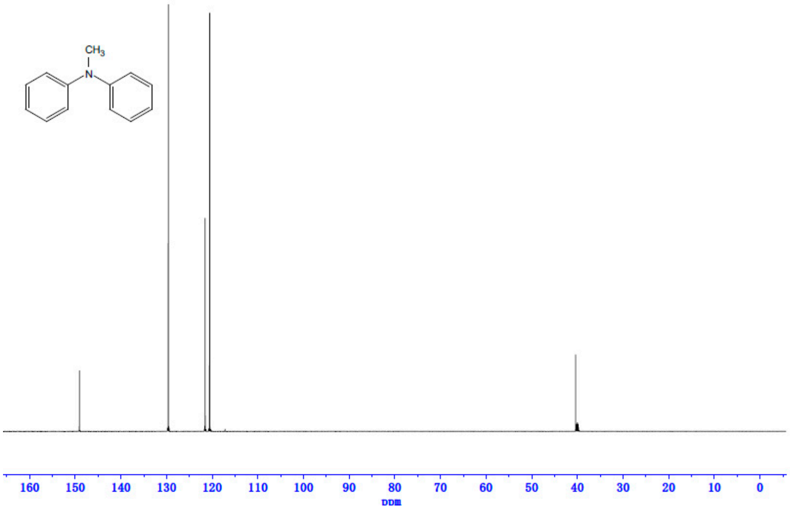

N-Methyldiphenylamine

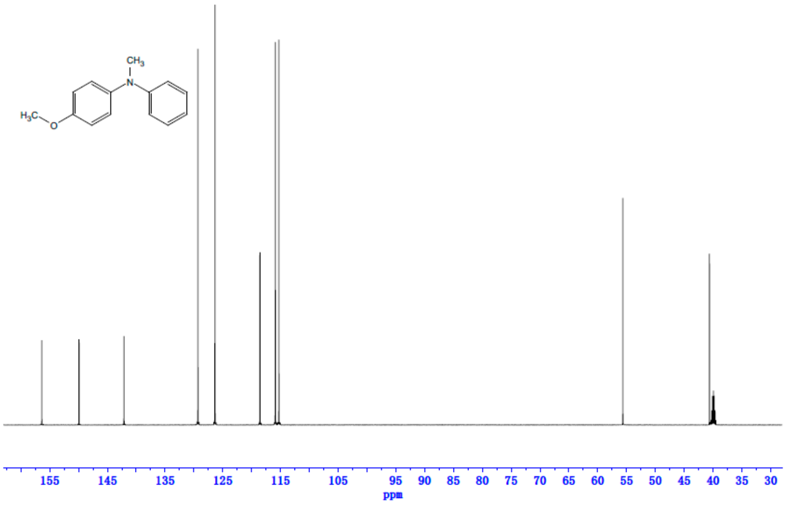

N-Methyl-N-(4-methoxy)phenylaniline

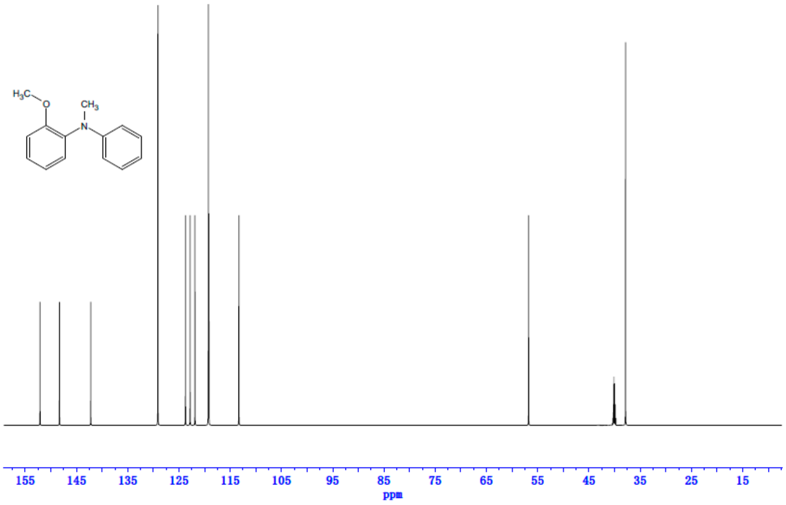

N-Methyl-N-(2-methoxy)phenylaniline

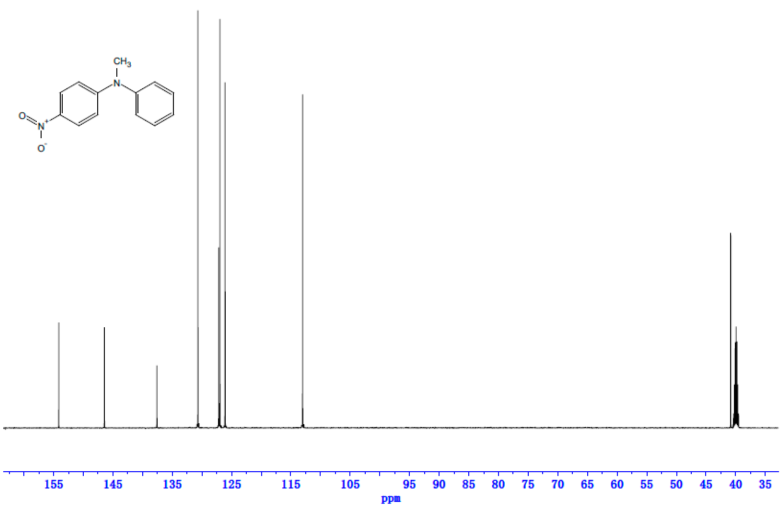

N-Methyl-N-(4-nitro)phenylaniline

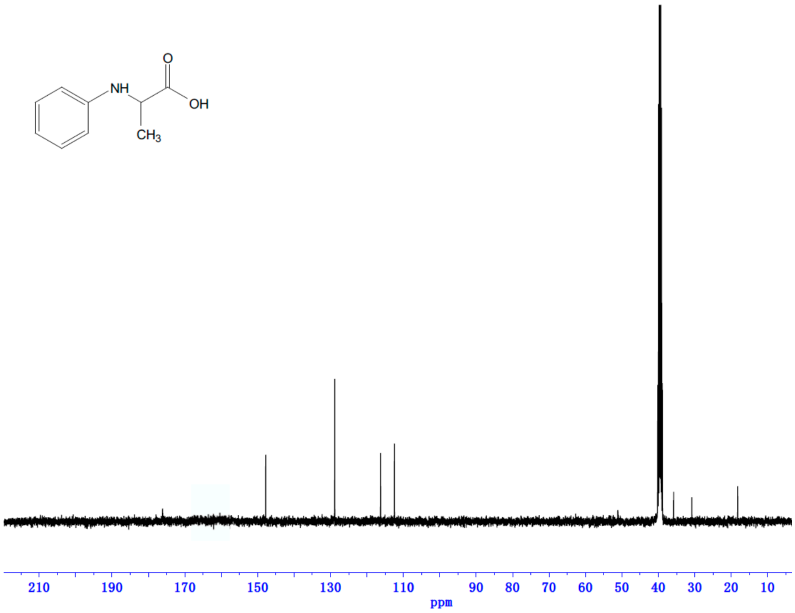

2-(Phenylamino)propanoic acid

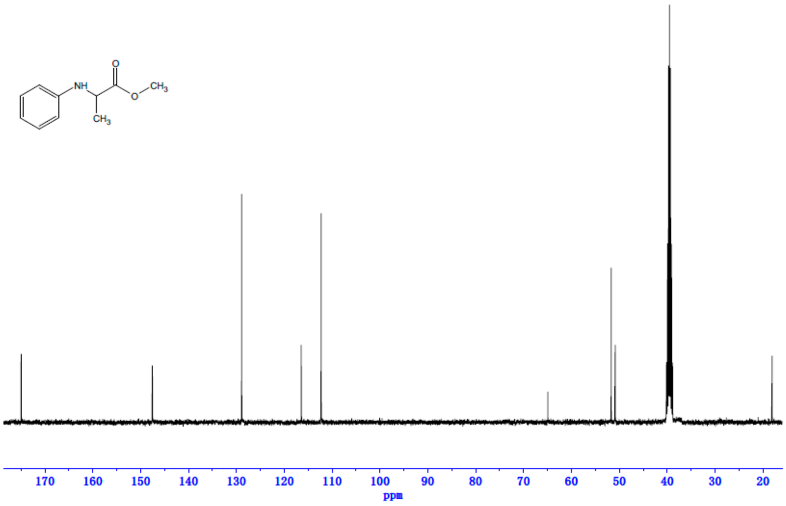

Methyl 2-(phenylamino)propanoate

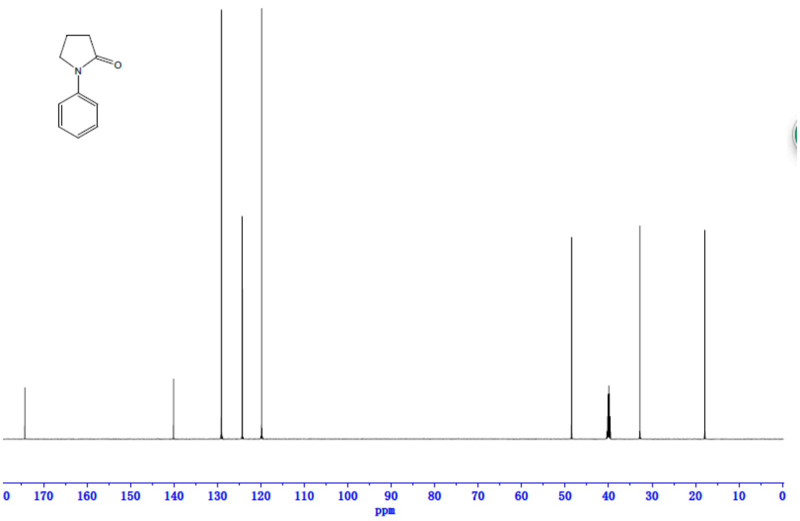

N-Phenyl-2-pyrrolidone

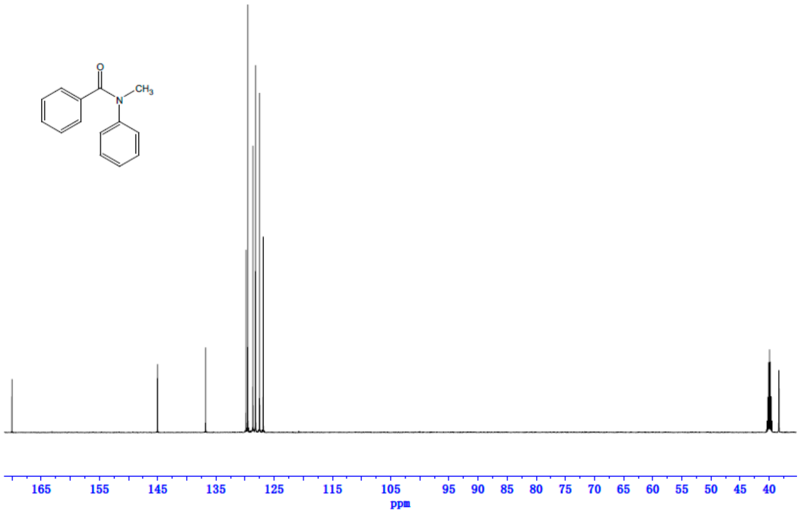

N-Methyl-N-benzoylaniline

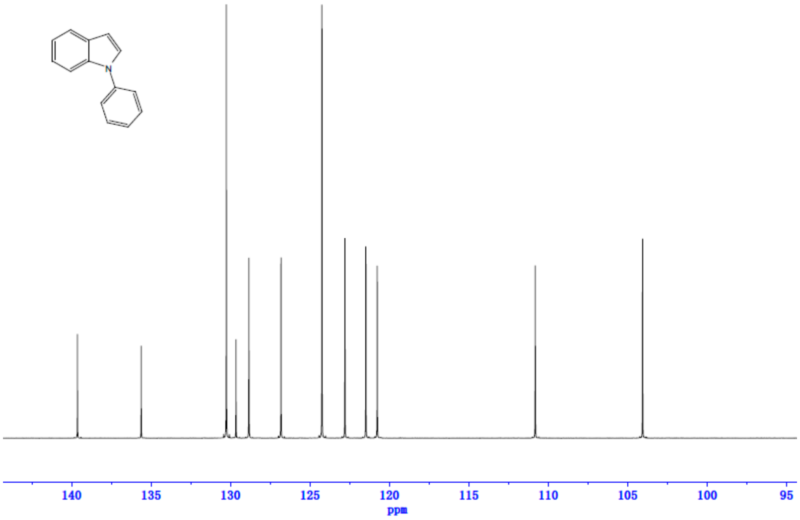

N-Phenylindole

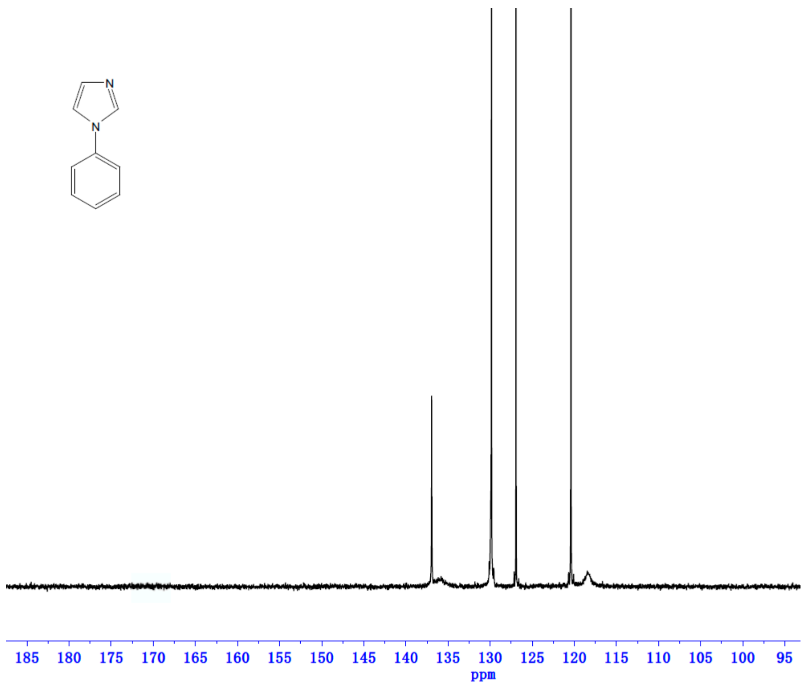

N-Phenylimidazole
